# Exocytosis of Progeny Infectious Varicella-Zoster Virus Particles via a Mannose-6-Phosphate Receptor Pathway without Xenophagy following Secondary Envelopment

**DOI:** 10.1128/JVI.00800-20

**Published:** 2020-07-30

**Authors:** James H. Girsch, Wallen Jackson, John E. Carpenter, Thomas O. Moninger, Keith W. Jarosinski, Charles Grose

**Affiliations:** a Division of Infectious Diseases/Virology, Children’s Hospital, University of Iowa, Iowa City, Iowa, USA; b Central Microscopy Research Facility, College of Medicine, University of Iowa, Iowa City, Iowa, USA; c Department of Pathobiology, College of Veterinary Medicine, University of Illinois at Champaign-Urbana, Urbana, Illinois, USA; University of California, Irvine

**Keywords:** Imaris, LAMP1, Pompe disease, Rab6, autophagosome, herpes simplex virus, pseudorabies virus

## Abstract

The literature on the egress of different herpesviruses after secondary envelopment is contradictory. In this report, we investigated varicella-zoster virus (VZV) egress in a cell line from a child with Pompe disease, a glycogen storage disease caused by a defect in the enzyme required for glycogen digestion. In Pompe cells, both the late autophagy pathway and the mannose-6-phosphate receptor (M6PR) pathway are interrupted. We have postulated that intact autophagic flux is required for higher recoveries of VZV infectivity. To test that hypothesis, we infected Pompe cells and then assessed the VZV infectious cycle. We discovered that the infectious cycle in Pompe cells was remarkably different from that of either fibroblasts or melanoma cells. No large late endosomes filled with VZV particles were observed in Pompe cells; only individual viral particles in small vacuoles were seen. The distribution of the M6PR pathway (*trans*-Golgi network to late endosomes) was constrained in infected Pompe cells. When cells were analyzed with two different anti-M6PR antibodies, extensive colocalization of the major VZV glycoprotein gE (known to contain M6P residues) and the M6P receptor (M6PR) was documented in the viral highways at the surfaces of non-Pompe cells after maximum-intensity projection of confocal z-stacks, but neither gE nor the M6PR was seen in abundance at the surfaces of infected Pompe cells. Taken together, our results suggested that (i) Pompe cells lack a VZV trafficking pathway within M6PR-positive large endosomes and (ii) most infectious VZV particles in conventional cell substrates are transported via large M6PR-positive vacuoles without degradative xenophagy to the plasma membrane.

**IMPORTANCE** The long-term goal of this research has been to determine why VZV, when grown in cultured cells, invariably is more cell associated and has a lower titer than other alphaherpesviruses, such as herpes simplex virus 1 (HSV1) or pseudorabies virus (PRV). Data from both HSV1 and PRV laboratories have identified a Rab6 secretory pathway for the transport of single enveloped viral particles from the *trans*-Golgi network within small vacuoles to the plasma membrane. In contrast, after secondary envelopment in fibroblasts or melanoma cells, multiple infectious VZV particles accumulated within large M6PR-positive late endosomes that were not degraded en route to the plasma membrane. We propose that this M6PR pathway is most utilized in VZV infection and least utilized in HSV1 infection, with PRV’s usage being closer to HSV1’s usage. Supportive data from other VZV, PRV, and HSV1 laboratories about evidence for two egress pathways are included.

## Abstract (precis)

The long-term goal of this research has been to determine why VZV, when grown in cultured cells, invariably is more cell associated and has a lower titer than other alphaherpesviruses, such as herpes simplex virus 1 (HSV1) or pseudorabies virus (PRV). Data from both HSV1 and PRV laboratories have identified a Rab6 secretory pathway for the transport of single enveloped viral particles from the *trans*-Golgi network within small vacuoles to the plasma membrane. In contrast, after secondary envelopment in fibroblasts or melanoma cells, multiple infectious VZV particles accumulated within large M6PR-positive late endosomes that were not degraded en route to the plasma membrane. We propose that this M6PR pathway is most utilized in VZV infection and least utilized in HSV1 infection, with PRV’s usage being closer to HSV1’s usage. Supportive data from other VZV, PRV, and HSV1 laboratories about evidence for two egress pathways are included.

## INTRODUCTION

A goal of this research has been to determine whether the autophagic flux pathway, which includes the late endosome, is relevant to varicella-zoster virus (VZV) egress after secondary envelopment. There are many conflicting reports in the herpes virology literature about the proviral and antiviral roles of autophagy in the infectious cycle (1–4). Likewise, there are many conflicting reports about the pathway (or pathways) by which herpesviruses are transported to the surface of an infected cell after secondary envelopment in the cytoplasmic virus assembly compartment (VAC) (5). Many investigators have selected various inhibitors of autophagy to gauge whether decreased autophagy has an effect on virus assembly (6). For example, we recently reported that the inhibitor of autophagic flux bafilomycin A1 (BAF) inhibited VZV secondary envelopment, confirming and expanding an earlier herpes simplex virus 1 (HSV1) observation (7, 8). Although widely used, inhibitors may exert unintended or unknown effects on cellular pathways that alter virus assembly (9–12). As an alternative strategy by which to study the effect of autophagy during herpesviral infection, we pursued a goal to find a human disease with a deficiency in the autophagy pathway. Often the deficiencies in the autophagy pathway have been well characterized in human diseases. Then we could use cells obtained from a patient with this genetic disease to assess the role of autophagy in the assembly pathway of a herpesvirus. In other words, if secondary viral assembly and egress proceeded without any impairment, then the autophagy pathway was not a component of the herpesviral assembly pathway. However, if the assembly or egress of a herpesvirus was limited by growth in this autophagy-deficient cell line, then the autophagy pathway may be a component of the herpesviral egress pathway.

After perusal of the recent literature on genetic deficiencies in human patients, we selected Pompe disease for further investigation. Pompe disease was described in 1932; it is a well-characterized but rare glycogen storage disease that often leads to an early death in afflicted children (13). Pompe disease is also known as glycogen storage disease II (GSD II). Pompe disease is caused by a mutation in the enzyme acid alpha-glucosidase (GAA), which limits its ability to hydrolyze glycogen to glucose in the lysosome (14). GAA is transported from the *trans*-Golgi network (TGN) to the late endosome via a mannose-6-phosphate receptor (M6PR) pathway, in which there is a prominent cation-independent M6PR (CI-M6PR) and a minor cation-dependent M6PR (CD-M6PR). Recently, investigators have discovered a deficiency in autophagic flux in Pompe disease (15, 16). For our virology studies, we selected the alphaherpesvirus VZV as the test pathogen because of related prior publications (8, 17). When we infected cells obtained from a child with Pompe disease, we observed during the first experiments that VZV infection and assembly were markedly decreased in Pompe cells compared with VZV infection and assembly in common cell substrates. Unexpectedly, after many additional infected Pompe cells were imaged by electron microscopy and hundreds of micrographs were examined, we also observed that the impaired VZV titers in Pompe cells correlated with a virtual absence of the most common egress pathway seen in VZV-infected fibroblasts or melanoma cells. When we compared our Pompe cell results with those of published VZV studies dating back several decades (18–20), we concluded that the Pompe cell studies had revealed the presence of two distinct egress pathways for progeny VZV particles following secondary envelopment and that the pathway observed in Pompe cells (small vacuoles) was (i) similar to pathways described for HSV1 and pseudorabies virus (PRV) but (ii) not the more common VZV egress pathway seen in conventional cell substrates (large vacuoles). A diagram of every pathway mentioned subsequently in Results is included within Fig. 10.

## RESULTS

### VZV infection and autophagy in Pompe cells.

**FIG 1:**
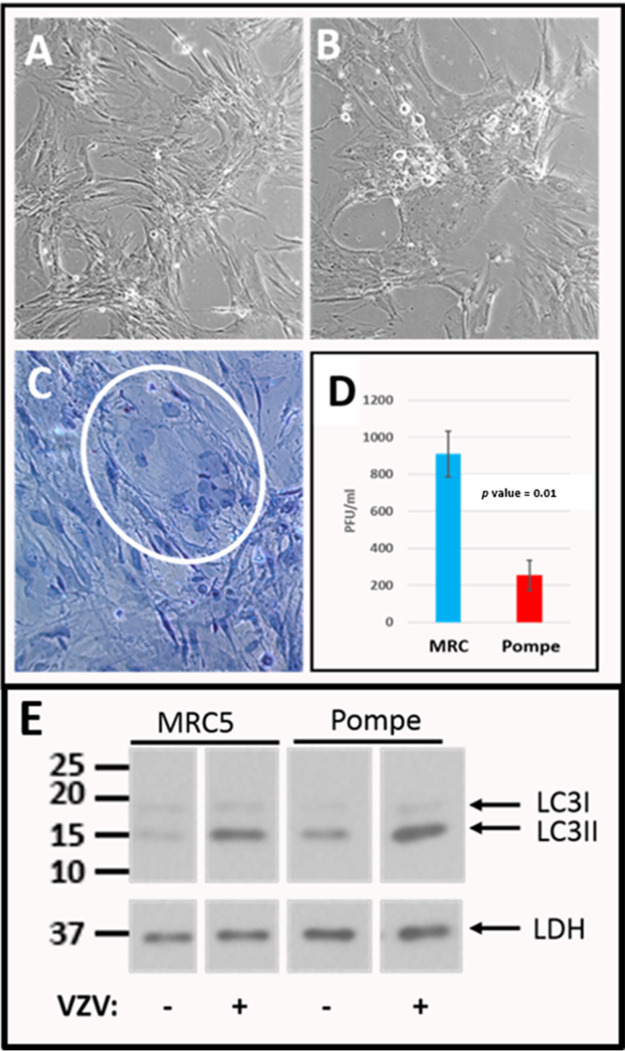
Images of Pompe cells before and after infection and viral titration. Pompe cells have been used in over 30 experiments. These cells divide more slowly than MRC-5 cells. (A, B) Pompe cells were initially examined by light microscopy before (A) and after (B) VZV infection. The monolayers never became completely confluent. (C) Infected Pompe cells were fixed with hematoxylin and eosin (H&E) staining and observed for foci of cytopathic effect. A small VZV-induced syncytium was visible (white circle). (D) VZV titer in infected Pompe cells compared with that in infected MRC-5 cells. Titration of cell-free VZV from an infected Pompe cell monolayer was measured by a plaque assay in MRC-5 cell monolayers, as described in Materials and Methods. Because Pompe cells never formed a fully confluent monolayer, we selected MRC-5 cell monolayers for infection that were at a similar stage of confluence. The assay was repeated three times after three separate infections. (E) Immunoblot for LC3-II. Lysates of Pompe cells and MRC-5 cells were immunoblotted with antibody to LC3, both before and after VZV infection. Lactate dehydrogenase (LDH) was the loading control. Numbers at the left are MWs, in kilodaltons.

Since autophagic flux mechanisms are reported to be deficient in Pompe cells, we hypothesized that viral titers would be low after VZV infection of Pompe cells (17). If there was no effect on virus titer, there would be no reason for our laboratory to continue to investigate VZV infection in Pompe cells. Since we found no prior publication about VZV infection of Pompe cells, we first performed a traditional infection and inspected the cells once daily for cytopathology. Before VZV infection, we noted that the Pompe cell monolayer never became completely confluent even after 7 days of incubation (Fig. 1A). Within 2 days postinfection (dpi) with an infected-cell inoculum, however, cytopathology was evident in the Pompe cell monolayers (Fig. 1B). The cytopathology was similar to that seen in infected MRC-5 cells but not as extensive; some clustering of nuclei was seen, suggestive of typical VZV-induced syncytium formation (Fig. 1C). As a positive control, we infected MRC-5 cell monolayers. Because Pompe cell monolayers never became fully confluent, we selected MRC-5 cell monolayers that had degrees of confluence similar to those of Pompe cell monolayers. When both sets of infected cell monolayers were harvested, we removed the medium and titrated the cells and the medium separately. We found a reduction in the VZV cell-free titer in an infected Pompe cell monolayer compared with that of an infected MRC-5 cell monolayer (Fig. 1D); we did not find cell-free infectivity in the medium of either set of monolayers. We also inoculated Pompe cells with cell-free virus; the cytopathology was the same as shown in Fig. 1A but required more days to appear.

Because of the importance of autophagy data for our proposed studies, we decided to confirm prior observations about increased microtubule-associated protein 1 light-chain 3-II (MAP1 LC3-II or LC3-II) in Pompe cells. We also wanted to repeat our original data about increased LC3-II after VZV infection of MRC-5 cells as a positive control, because another laboratory had not found a similar response (21). When performing this experiment in MRC-5 cells, we allowed the cells to become confluent before infection (Fig. 1E). At that time, there was little or no LC3-I or LC3-II detectable, as expected in quiescent cells with an intact autophagic pathway (Fig. 1E, MRC5, VZV – lane); within 72 h postinfection (hpi), there was an obvious increase in LC3-II levels (Fig. 1E, MRC5, VZV + lane), as previously documented following the stress response to VZV infection (22). On the other hand, there was more LC3-II present in the uninfected Pompe cells than in uninfected MRC-5 cells because of a known block in autophagic flux in Pompe cells (Fig. 1E, Pompe, VZV – lane). After VZV infection, there was a further increase in LC3-II in Pompe cells, which indicated an additive effect of a stress response after infection in cells with a block in autophagic flux (Fig. 1E, Pompe, VZV + lane). In summary, we confirmed prior data about higher LC3-II levels in uninfected Pompe cells and added new data about the stress response of Pompe cells to virus infection.

### Confocal microscopic examination of uninfected and infected Pompe cells.

**FIG 2:**
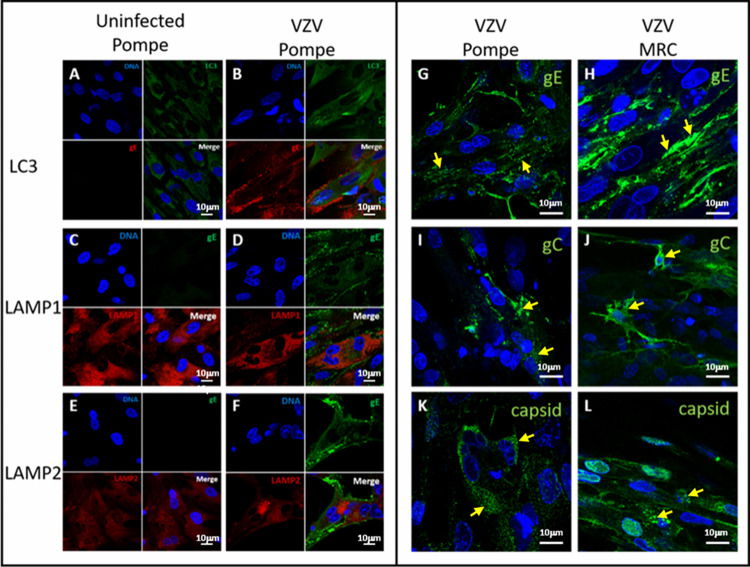
Imaging of uninfected and infected Pompe cell monolayers by confocal microscopy. Uninfected Pompe cells were probed for LC3 (A), LAMP1 (C), and LAMP2 (E). Infected Pompe cells were probed for LC3 (B), LAMP1 (D), and LAMP2 (F). Individual and merged channels are shown in panels A to F. Infected Pompe cells were probed for VZV gE (G), gC (I), and capsid (ORF 41) (K). As a positive control, infected MRC-5 cells were probed for VZV gE (H), gC (J), and capsid (L). Panels G to L contain yellow arrows to mark the viral protein of interest. Only the merged channel is shown in panels G to L.

Because of our extensive autophagy studies during VZV infection using confocal microscopy (23), we also wanted to confirm the dysregulation of autophagy in both uninfected and infected Pompe cells by confocal microscopy. We found increased LC3-II in Pompe cells, reflecting an inhibition of autophagic flux (Fig. 2A). This increase in LC3-II expression correlated with the increased LC3-II observed by immunoblotting (Fig. 1). We also observed by confocal microscopy the increased distribution of LC3-II in Pompe cells after infection and the presence of the characteristic LC3-II puncta (Fig. 2B). Finally, we chose to look at lysosome-associated membrane proteins 1 and 2 (LAMP1 and LAMP2) in these cells (24). Our LAMP1 and LAMP2 immunolabeling results from uninfected cells corresponded with published observations and thereby confirmed that our Pompe cell line had features representative of features published previously (Fig. 2C and E). We also documented LAMP1 and LAMP2 expression after VZV infection (Fig. 2D and F).

Next, we wanted to compare levels of viral protein expression in Pompe cells and MRC-5 cells by confocal microscopy as a confirmation of the decreased VZV titer in Pompe cells. For this experiment, we selected the VZV structural glycoprotein gE, which is the major type 1 VZV glycoprotein expressed in the early-late phase of the VZV replication cycle (25). By confocal microscopy, we showed a markedly different trafficking pattern for gE in Pompe cells; specifically, gE was found in cytoplasmic membranes, but only minimal amounts of gE were found on the outer cell membrane (Fig. 2G). In contrast, in MRC-5 or melanoma cells, expression of gE was much more widespread, especially on the cell surface, where gE outlined linear rows of egressing viral particles that we have called viral highways (Fig. 2H) (26).

We also examined the monolayers for a VZV capsid protein (open reading frame 41 [ORF 41]) and the VZV gC protein, a true late protein (27). Expression of the gC glycoprotein was detected in Pompe cells, but as previously seen with gE, the expression was considerably reduced and not apparent on the surfaces of infected cells (Fig. 2I and J). The distributions of the capsid were different between Pompe cells and MRC-5 cells. In Pompe cells, there was a punctate appearance across the cytoplasm (Fig. 2K), while in MRC-5 cells, there were larger collections within vacuole-like structures in the cytoplasm (Fig. 2L). Since both the capsid protein and the gC glycoprotein were detected, this was an indication that VZV infection in Pompe cells proceeded through all three phases of the replication cycle and that complete viral particles were assembled. However, the minimal amount of immunolabeling for VZV gE on the surfaces of VZV-infected Pompe cells strongly suggested that fewer viral particles were transported to the outer cell membrane, unlike with VZV-infected MRC-5 cells (Fig. 2G and H).

### Comparison of uninfected Pompe cells with uninfected MRC-5 cells and uninfected BAF-treated MRC-5 cells.

**FIG 3:**
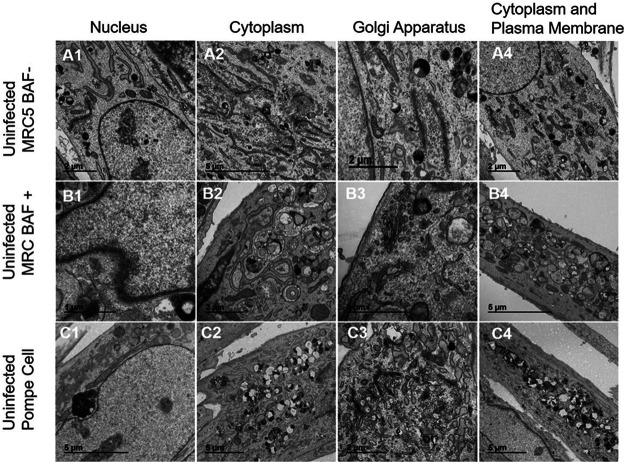
Examination of uninfected Pompe cells by electron microscopy. To define intracellular abnormalities within Pompe cells before viral infection, these cells were imaged by TEM and compared with untreated and uninfected MRC-5 cells as well as MRC-5 cells treated with an inhibitor of autophagy, BAF. (A1 to A4) Uninfected and untreated MRC-5 cells. (B1 to B4) BAF-treated uninfected MRC-5 cells. (C1 to C4) Uninfected Pompe cells. Differences between MRC-5 cells and either BAF-treated cells or Pompe cells are discussed in Results. Note the absence of a thickened nuclear membrane in panel C1.

Because we planned to observe infected Pompe cells by transmission electron microscopy (TEM) in order to investigate VZV trafficking pathways, we first needed to observe uninfected Pompe cells by TEM, in part because we were unable to find sufficient examples in the literature. As a control, we reexamined uninfected MRC-5 cells, and as a second control for autophagy inhibition, we reexamined MRC-5 cells treated with BAF. We divided the micrographs into images of four areas of the cell: nucleus, cytoplasm, Golgi apparatus, and cytoplasm with plasma membrane. Compared with uninfected and untreated MRC-5 cells (Fig. 3A1 to -4), BAF-treated MRC-5 cells exhibited slightly thickened nuclear membranes (Fig. 3B1 to -4). BAF treatment was an important control for Pompe cells, because BAF blocks almost completely the transition from autophagosome to autophagolysosome in normal cells (28). Therefore, there was an accumulation of autophagosomes in the cytoplasm, as well as distinctive multivesicular bodies (Fig. 3B2 to -4). When we compared uninfected Pompe cells (Fig. 3C1 to -4) to the two above-mentioned sets of micrographs, we noticed some similarity with TEMs of BAF-treated MRC-5 cells. In particular, throughout the cytoplasm of Pompe cells, there was a marked accumulation of multivesicular bodies, in addition to numerous distinctive large prelysosomes filled with electron-dense material known to be glycogen. The Golgi apparatus in Pompe cells was similar to that in MRC-5 cells.

### Comparison of the nuclei of infected Pompe cells with those of infected MRC-5 cells and infected BAF-treated MRC-5 cells.

**FIG 4:**
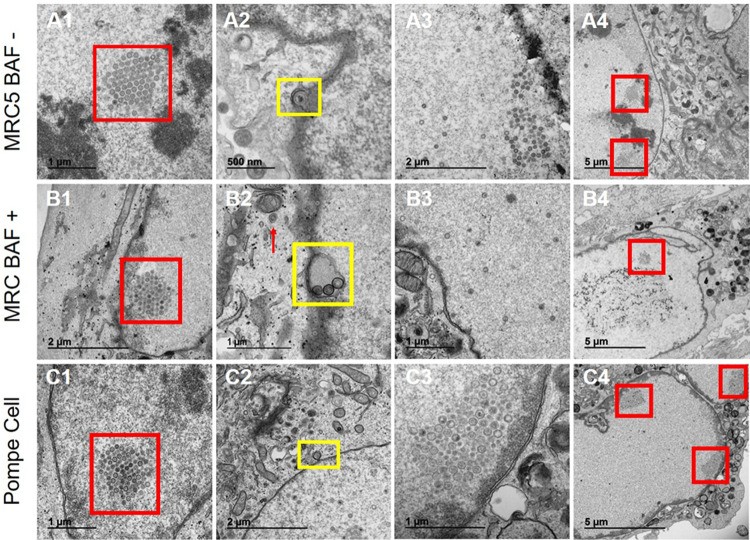
Nucleocapsid assembly and nuclear egress under three conditions of VZV infection. (A) MRC-5 cells not treated with BAF after infection. Capsids were easily detected in the nucleus. (A1) A latticework of capsids was observed in some nuclei (red box). (A2) Nuclear egress (yellow box). (A3) Nucleoplasm. (A4) Latticework of capsids (red boxes). (B) MRC-5 cells treated with BAF after infection. (B1) Latticework of capsids (red box). (B2) Nuclear egress. Three capsids are clustered in one compartment derived from the inner nuclear membrane (yellow box); there is an unenveloped capsid in the cytoplasm (red arrow). (B3) Nucleoplasm. (B4) Capsids (red box). (C) Pompe cells after VZV infection. (C1) Latticework of capsids (red box). Note the similarity in panels A1 and B1. (C2) Nuclear egress (yellow box). (C3) Nucleoplasm. (C4) Capsids (red boxes).

Next, we examined the nuclei of infected Pompe cells in comparison with the nuclei of infected MRC-5 cells and infected BAF-treated MRC-5 cells. We reasoned that an impairment in VZV nuclear egress in Pompe cells may signal an unanticipated alteration in VZV capsid formation and tegumentation and thereby compromise our ability to directly compare means of egress after secondary envelopment in the cytoplasm. Capsid formation under typical conditions in infected MRC-5 or human melanoma cells is known to lead to aberrant capsids, many of which lack a DNA core (29, 30). As shown in untreated MRC-5 cells (Fig. 4A1 to -4), capsid formation frequently led to a latticework of capsids in the nucleus. Capsids exiting the nuclear membrane were easily detected; unlike with HSV1, many VZV capsids with aberrant cores transit the inner and outer nuclear membranes (31). There were few differences between the appearance of the capsid in the nucleus or the nuclear membrane of infected Pompe cells and that of BAF-treated MRC-5 cells (Fig. 4B1 to -4). The structures of the capsids were similar to those of capsids in MRC-5 cells, with a variety of phenotypes that have been previously documented by this laboratory (30). For example, many of the capsids lacked a DNA core (Fig. 4C1 to -4). The above-described results indicated no apparent block in capsid formation in and egress from the nuclei of Pompe cells.

### Different viral pathways in the cytoplasm of infected Pompe cells.

**FIG 5:**
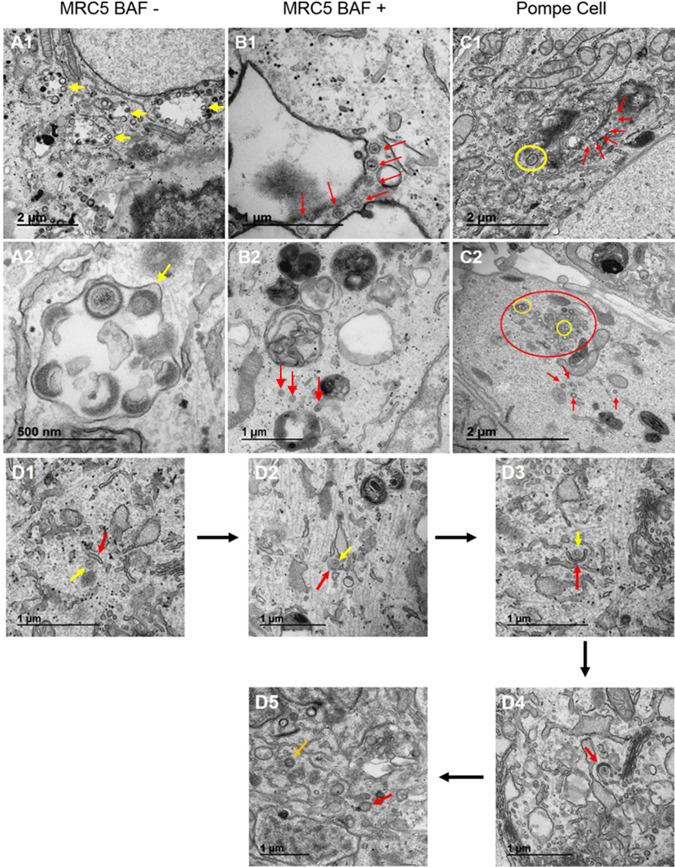
Cytoplasm of VZV-infected cells under three different conditions. (A) Cytoplasm of VZV-infected MRC-5 cells. (A1) Large vacuoles in the cytoplasm, each containing several viral particles (yellow arrows). (A2) Higher magnification of a large vacuole containing at least 6 viral particles, one of which is prototypic, while others are aberrant (yellow arrow). (B) Cytoplasm of bafilomycin-treated VZV-infected MRC-5 cells. (B1) Large empty vacuoles with a few adjacent capsids (red arrows). (B2) Capsids in the cytoplasm adjacent to vacuoles (red arrows). Multivesicular bodies are also seen, a common feature after BAF treatment. (C) Pompe cells after VZV-induced cytopathology was evident. (C1) Multiple capsids in the cytoplasm and one viral particle within a single vesicle (circle). (C2) Many capsids clustered together in the cytoplasm (red circle) together with a few wrapping membranes near a few capsids (yellow circles). (D) Progression of capsid wrapping in VZV-infected Pompe cells. (D1 to D4). Wrapping membranes at various stages. A yellow arrow designates the capsid, and a red arrow designates the wrapping membrane. (D5) Nearly completely wrapped capsid. A red arrow designates a wrapping membrane that almost completely surrounds a capsid, and an orange arrow designates a completely enclosed capsid.

Because the titer of virus recovered from infected Pompe cells was low, we predicted that we would see differences in virus assembly and egress in the cytoplasm of Pompe cells as visualized by TEM. When we observed all three conditions of infection by TEM, we noticed marked differences in the cytoplasm after the three infections (Table 1). The data in Table 1 represent observations from 483 new micrographs. Two differences are highlighted: (i) small or large vacuoles containing viral particles and (ii) capsids or no capsids in the cytoplasm. In untreated infected MRC-5 cells, clusters of viral particles, including both light particles and virions, were usually found in larger vacuoles. Naked capsids were difficult to find. Viral particles were easily located in vacuoles (Fig. 5A1 and -2). In BAF-treated MRC-5 cells, naked capsids and light particles within vacuoles were observed, but enveloped complete virions were rarely seen. Light particles were occasionally seen in the vicinity of the plasma membrane (Fig. 5B1 and -2). By comparison, after virus infection of Pompe cells, we noticed many capsids in the cytoplasm, some of which were enclosed in small vacuoles. The cluster of capsids seen in one micrograph was the greatest number of capsids that we have ever seen in the cytoplasm of an infected cell (Fig. 5C2). At this point, we reexamined our large archive of electron micrographs from past experiments and confirmed that even one or two naked capsids within a single micrograph were very difficult to find in the cytoplasm of VZV-infected MRC-5 or human melanoma cells (29). Further, we noted in our prior paper that naked capsids were sometimes in the cytoplasm of BAF-treated MRC-5 cells, often surrounding but not within vacuolar compartments (8). However, the number was fewer than that seen in VZV-infected Pompe cells. During our examination of the 483 new micrographs, we found 5 that provided examples of the evolving process of wrapping a capsid in Pompe cells (Fig. 5D1 to -5). What we have designated a wrapping membrane was similar in appearance to what other virologists have called wrapping membranes derived from the *trans*-Golgi network (32). We also note that the vacuoles containing single viral particles were too wide in diameter (200 to 250 nm) to be exosomes. Even though we found wrapped viral particles in the cytoplasm, very few particles were seen along the plasma membrane in infected Pompe cells. Finally, we did not observe xenophagy of viral particles under any of the three conditions of infection (Table 1).

**TABLE 1:** Comparison of major findings for VZV-infected cells by electron microscopy

Compartment	Finding for:		
	MRC-5 cells	BAF–MRC-5 cells	Pompe cells
Nucleus	Many capsids	Many capsids	Many capsids
Nuclear membrane	Egress through nuclear membrane	Egress through thickened membrane	Similar to MRC-5
Cytoplasm	Few capsids	More capsids	Many more capsids
	Several large vacuoles with viruses	Occasional vacuoles with light particles	Few small vacuoles with a single virus each
Golgi apparatus	Normal	Fragmented	Relatively normal
Lysosomes	No virus	No virus	No virus
Plasma membrane	Many viral particles	Few light particles	Few viral particles

### Density gradient sedimentation of VZV-infected Pompe cells.

**FIG 6:**
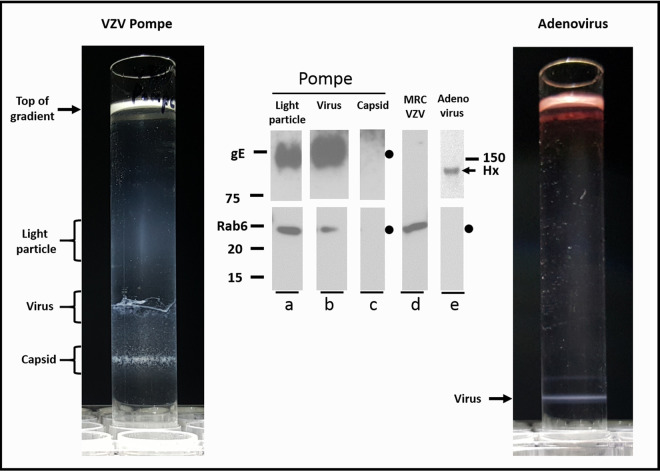
Purification of virus from Pompe cells by density gradient sedimentation. Monolayers of VZV-infected Pompe cells were harvested by procedures described in Materials and Methods and then subjected to density gradient sedimentation to purify light particles and viral particles (left side). After completion of sedimentation, the gradient was photographed and the individual bands were removed for analysis by immunoblotting. As a control for VZV particles purified by gradient sedimentation, adenovirus was also purified in identical sedimentation gradients and photographed, and the single viral band was removed for analysis by immunoblotting (right side). (Blot a) VZV light particle band immunoblotted for gE and Rab6. Molecular weight markers are included alongside this lane. (Blot b) VZV virus band immunoblotted for gE and Rab6. (Blot c) VZV capsid band immunoblotted for gE and Rab6. (Blot d) VZV-infected cell lysate immunoblotted for Rab6. (Blot e) Adenovirus band immunoblotted for viral hexon (Hx) and Rab6.

Based on the differences in infected Pompe cells (single vacuoles, each with one viral particle) versus infected MRC-5 cells (large vacuoles with many viral particles), we realized that we had found a cell line with a restricted egress pathway never seen before in any other cell line examined by our laboratory since we first looked in 1978 (33). Our hypothesis for the next set of experiments was based on data collected by PRV investigations (34). They have shown that complete PRV virions egress via a pathway identified by the Rab6 protein (34, 35). Therefore, we predicted that the egress pathway for VZV in infected Pompe cells was the same pathway, since VZV particles were transported in the cytoplasm as single viral particles in small vacuoles. When we sedimented a lysate of VZV-infected Pompe cells in our well-characterized density gradients, we observed three bands (Fig. 6). The uppermost two bands were in the usual locations for the light particle band and the enveloped virus band; the third band was lower in the gradient (Fig. 6). When we performed immunoblotting on each band, the two uppermost bands were positive for the gE protein, an indication that they represented the light particle band and the enveloped virus band, as expected (Fig. 6, blots a and b). The lowermost band, which lacked the gE protein, was the capsid band (Fig. 6, blot c). When the light particle and virus bands were subjected to immunoblotting with Rab6 antibody, the result was positive. The capsid band was negative for Rab6. To confirm the specificity of our gradient fractionation, we intentionally overexposed the gE immunoblot (Fig. 6, blots a to c) in order to show that the lowermost band was indeed gE negative and that trace amounts of the VZV gE protein were not found outside the light particle and virus bands (Fig. 6, blot c). The VZV-infected cell lysate was probed for Rab6 without undergoing gradient sedimentation (Fig. 6, blot d). We included the entire length of the gel to show the lack of nonspecific reactivity by the anti-Rab6 reagent to other proteins.

As another control for the specificity of our gradient sedimentation, we also purified adenovirus in the same density gradient. Adenovirus is another double-stranded DNA virus, but adenovirus lacks an envelope (80 nm in diameter). As can be seen, a single band was detected after gradient sedimentation at approximately the same location as that of the VZV capsid band in the Pompe sedimentation (Fig. 6, compare left and right sides). This band immunoblotted with the hexon antibody (Fig. 6, blot e). Finally, we tested the adenovirus band for the Rab6 protein, but the Rab6 protein was not present (Fig. 6, blot e). This result indicated that the Rab6 protein did not bind nonspecifically to any virus; the result also indicated that trace amounts of the Rab6 protein were not found nonspecifically throughout this density gradient, even though Rab6 was easily detectable in the cells in which adenovirus was propagated (Fig. 6, blot d).

### Colocalization of M6PR and VZV gE during VZV infection documented by two-dimensional (2D) and 3D imaging.

We have previously proposed that VZV exits the cell by two different pathways, because the results about virus egress published by several laboratories (including our laboratory) over the past decades could not be explained by egress in a single known cellular pathway (36). When we reviewed all the above results from VZV-infected Pompe cells, we concluded again that there appeared to be two pathways by which VZV could egress to the cell surface after secondary envelopment: one pathway that was blocked in Pompe cells but common in MRC-5 or human melanoma cells (egress of multiple viral particles in larger vacuoles) and another that was not blocked in Pompe cells (egress of single viral particles in a small vacuole). In human cells, the enzyme GAA is carried from the TGN to the late endosome in a pathway mediated by the M6PR; in Pompe cells, the M6PR pathway is greatly impaired with subsequent accumulation of undigested glycogen in the late endosomes. We were also aware of prior investigations by both an HSV1 laboratory and a VZV laboratory that had proposed a role for the M6PR in HSV1 and VZV trafficking (37–39). Because of these accumulating data about M6PR functions and the knowledge that M6PR trafficking was greatly impaired in Pompe cells, we examined the localization of the M6PR in all cells and under all conditions previously described above.

**FIG 7:**
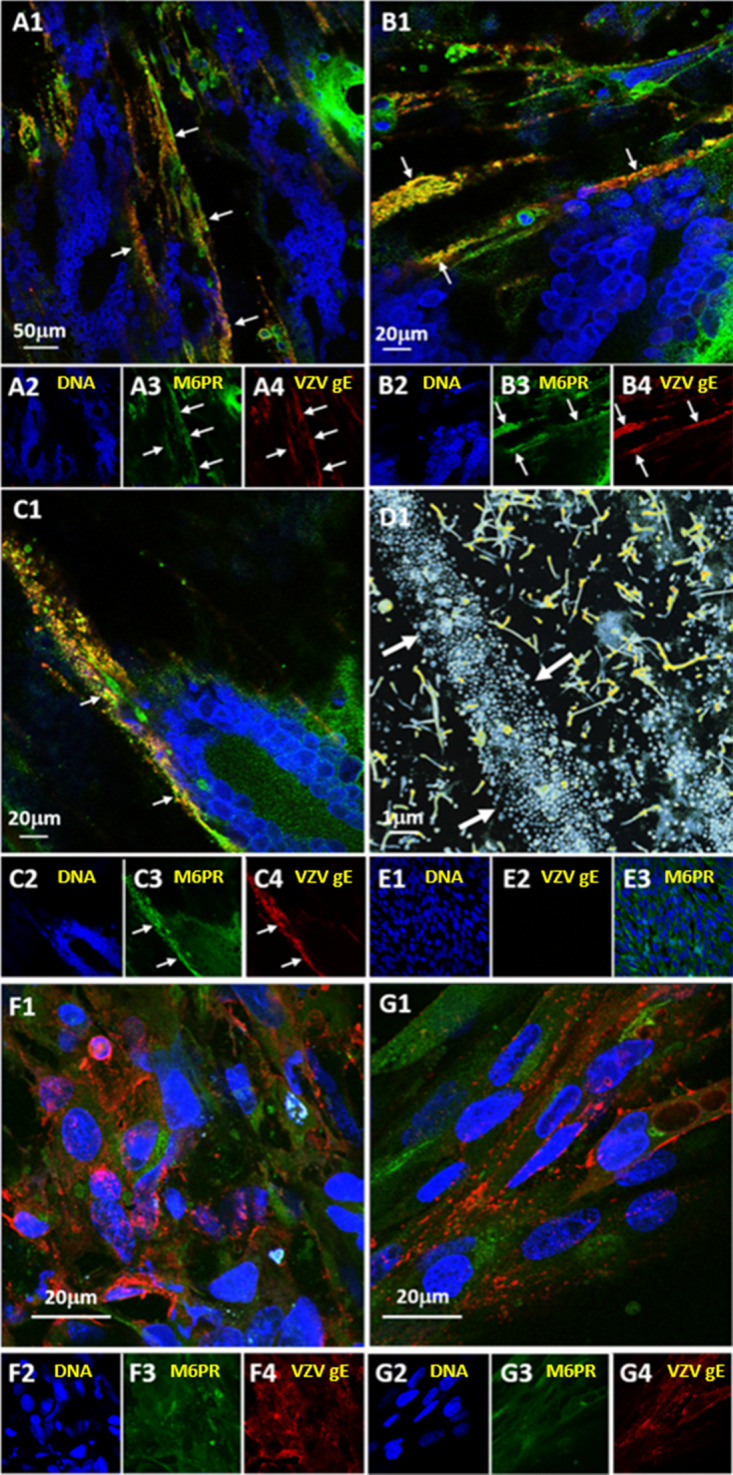
VZV gE and M6PR colocalization on the surface of a VZV-infected monolayer. VZV-infected human melanoma cells or Pompe cells were labeled with mouse anti-VZV gE MAb 3B3, followed by incubation with goat anti-mouse secondary antibody conjugated to Alexa Fluor 546 (red) and rabbit anti-M6PR polyclonal antibody and then by incubation with goat anti-rabbit secondary antibody conjugated to Alexa Fluor 488 (green). Nuclei were labeled with Hoechst 33342 stain (blue). (A to D) VZV-infected melanoma cells. Images selected from 3 of 4 separate experiments. (A1 to C1) Maximum-intensity projections showing labeled nuclei, VZV gE, and M6PR. (A2 to C2) Maximum-intensity projections of individual channels showing labeled nuclei. (A3 to C3) Maximum-intensity projections of individual channels showing labeled M6PR. (A4 to C4) Maximum-intensity projections of individual channels showing labeled VZV gE. White arrows indicate VZV gE and M6PR colocalization in the viral highways. (D1) Scanning electron micrograph of a viral highway. The micrograph shows the surface of a VZV-infected cell with hundreds of viral particles on the plasma membrane aligned in a viral highway (arrows). Viral particles are pseudocolored blue, and extensions from the cell surface are pseudocolored yellow. (E) Uninfected monolayer. (E1 to E3) Confocal images of an uninfected cell control monolayer. (E1) Stain for nuclei only. (E2) Immunolabel for VZV gE only (negative control). (E3) Merge of immunolabel for the M6PR and stain for nuclei. (F and G) VZV-infected Pompe cells. (F1 and G1) Merged images. (F2 to F4 and G2 to G4) Individual channels. Images were selected from 2 of 4 separate experiments. Note the absence of viral highways on the surfaces of infected cells in panels F1 and G1, compared with those in panels A1 to C1.

We have observed in prior studies that a greater number of viral particles are produced in human melanoma cells than in MRC-5 cells and that the viral particles egress onto the cell surface in a distinctive pattern called viral highways, although particles are not released in the medium (29). Therefore, we reasoned that any virus-M6PR interaction would be easier to detect in human melanoma cells because the viral highways are easily seen by confocal microscopy. We performed the next confocal microscopy experiments under both permeabilized and nonpermeabilized conditions with each of two anti-M6PR antibodies obtained from two different sources (40, 41); we found colocalization of gE with the M6PR under all 4 conditions. We included 3 representative confocal micrographs, each with 3 individual panels and 1 larger merged panel (Fig. 7A to C). We chose maximum-intensity projection after we reduced confocal z-stacks into individual 2D images. We observed an abundant expression of the M6PR in the cytoplasm as well as on the cell surface (Fig. 7A to C). Furthermore, we noted colocalization of the M6PR with VZV gE on the plasma membrane; at this location, the antibody detects gE that is present on viral particles within viral highways that include thousands of viral particles that have emerged onto the cell surface but never detach. We also included a scanning electron micrograph to show the viral highways extending in a linear configuration over surface areas of multiple infected cells that have fused because of the VZV fusogenic complex (Fig. 7D1). As a control, we observed the M6PR in uninfected melanoma cells, where the M6PR was found mainly in the Golgi complex surrounding the nuclei (Fig. 7E1 to -3). The immunolabeling patterns were different in infected Pompe cells. As noted in Fig. 2, although gE was synthesized in infected Pompe cells, viral infection of Pompe cells did not lead to large foci of gE immunolabeling on the plasma membrane, nor were viral highways detected (Fig. 7F and G). Scattered foci of gE and M6PR immunolabeling were detected in the cytoplasm, but there was little or no colocalization of gE and the M6PR.

**FIG 8:**
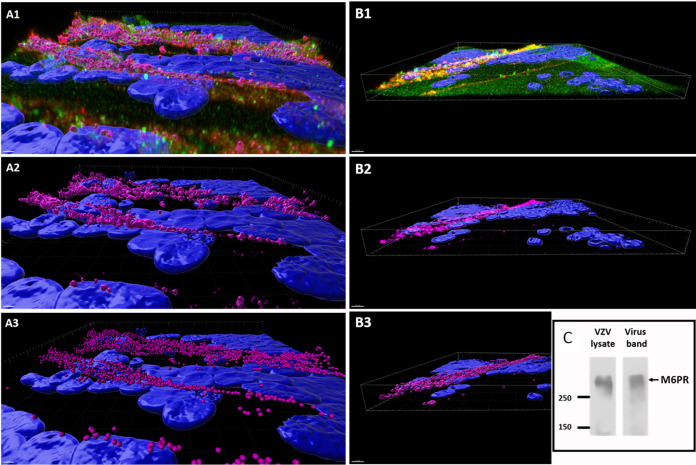
Imaris 3D confocal renderings of immunolabeled VZV-infected MeWo cells and the M6PR. Human melanoma cells were VZV infected, immunofluorescently labeled for VZV gE protein (red) and the M6PR (green), and imaged by confocal microscopy under both permeabilizing and nonpermeabilizing conditions. We carried out four separate imaging experiments and selected 11 z-stacks for further analysis by Imaris software. (A, B) We chose two examples from the 11 z-stacks. All images include isosurface renderings of stained nuclei (blue). (A1) Maximum-intensity projections of viral protein (red) and the receptor (green). Regions of colocalization are identified by magenta isosurface renderings. For images shown in panels A2 and A3, all noncolocalizing voxels were removed. (A2) Isosurface renderings of virus and receptor colocalization. (A3) Regions of virus and the receptor colocalization detected in the Imaris Spots module. (B1) Maximum-intensity projections of viral protein (red) and the receptor (green) and regions of colocalization (yellow). For images shown in panels B2 and B3, all noncolocalizing voxels were removed, and the colocalized proteins were colored magenta. (B2) Maximum-intensity projections of regions of virus and receptor colocalization identified with the color magenta under nonpermeabilized conditions. (B3) Isosurface renderings of regions of virus and receptor colocalization under nonpermeabilized conditions. (C) Immunoblot of M6PR. VZV was purified from infected MRC-5 monolayers by density gradient sedimentation. The infected cell lysate before sedimentation and the purified virus band from fraction 12 in the sedimentation gradient were probed for the M6PR.

To expand the spatial organization of the organelles within the images, we also analyzed 11 sets of z-stacks (each z-stack contains about 40 images) with Imaris software, which converts each z-stack into a 3D animation, allowing the viewer to see the depth within a cell where the virus-organelle interaction of interest occurs. To this end, each confocal z-stack was analyzed with Imaris 3/4D Image Visualization and Analysis software; the Imaris Coloc module was used to identify colocalization of virus and the M6PR. The areas of colocalization were displayed as both maximum-intensity projections and isosurface renderings (Fig. 8). The length of the viral highway shown in Fig. 8A1 was 135 μm; colocalization of gE and the M6PR was readily apparent. In Fig. 8A3, colocalization was represented by Spots Creation within Imaris software. Spots function converts similarly sized foci of colocalization into small colored circles. The length of the viral highway shown in Fig. 8B1 was 210 μm. Because the viral highways are localized at the surfaces of infected cells, we observed little difference in the profiles of the viral highways when the monolayers were examined under either permeabilized or nonpermeabilized conditions. Compared with the 2D images in Fig. 7, the representations of the 3D animations clearly affirmed the colocalization of the gE and M6PR proteins at or near the surface of the infected monolayer, within the linear pattern of viral highways (Fig. 8).

We carried out the above-described confocal colocalization experiments in VZV-infected melanoma cells because of the distinctive viral highways on the cell surface. As a further positive control, we purified VZV from infected MRC-5 cells by density gradient sedimentation. The infectious virus band was collected from fraction 12 of the gradient and subjected to immunoblotting with rabbit anti-M6PR antibody. The VZV-infected cell lysate prior to sedimentation was added to the immunoblot experiment. As can be seen in Fig. 8C, the M6PR copurified with the infectious virus band from VZV-infected MRC-5 cells. The M6PR protein migrated to a location (∼275 kDa; CI-M6PR) similar to that shown in Fig. 2 in the paper from the Johnson laboratory, which originally produced and characterized this anti-M6PR antibody reagent (41).

### Effect of VZV infection on transcription of CI-M6PR or CD-M6PR in MRC-5 cells or Pompe cells.

**FIG 9:**
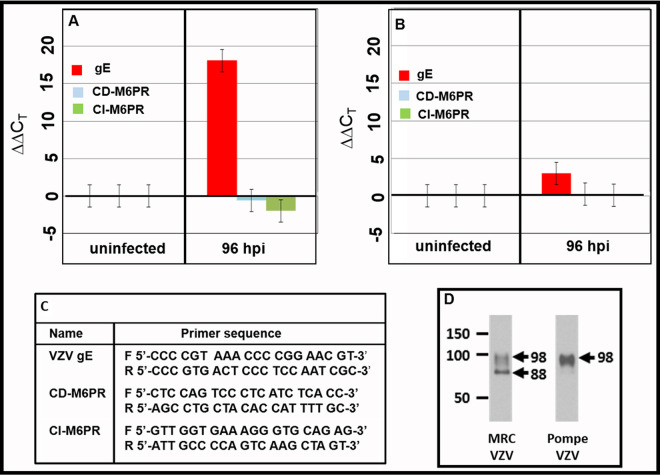
Transcription analysis for VZV gE and M6PR in VZV-infected MRC-5 and Pompe cells. Total RNA was isolated from cells and converted to cDNA, and then RT-PCR was performed with specific primers to VZV gE and cellular CD-M6PR and CI-M6PR. The *C_T_* values were normalized to glyceraldehyde 3-phosphate dehydrogenase (GAPDH), and then values relative to those of uninfected cells were computed. Compared with RNA from uninfected cells, CD-M6PR and CI-M6PR transcripts were largely unchanged in VZV-infected cells. (A) MRC-5 cells; (B) Pompe cells; (C) primers; (D) immunoblotting of VZV gE.

After investigating the expression of the M6PR protein, we also examined transcription representing the receptor in uninfected and VZV-infected cells. To this end, we carried out real-time (RT)-PCR measurements of RNAs for VZV ORF 68 (VZV gE) together with loci encoding both forms of the M6PR in MRC-5 cells and Pompe cells. VZV infection of MRC-5 cells was robust by 72 to 96 hpi, as indicated by high levels of expression of VZV gE RNA, although both CD-M6PR and CI-M6PR RNA levels were not significantly changed (Fig. 9A). VZV infection of Pompe cells exhibited a lower expression of VZV gE RNA, with little or no change in the levels of either M6PR transcript at 96 hpi (Fig. 9 B).

We also immunoblotted the gE protein in order to verify the integrity of glycoprotein processing in infected Pompe cells, because of the reduced transcription compared to that of the conventional cell substrate (42). The fully glycosylated mature gE protein has a molecular weight (MW) of 98 KDa. We discovered that the gE glycoprotein was fully mature in Pompe cells, with fewer lower-MW forms than in MRC-5 cells. Based on detailed studies of VZV gE processing carried out by this laboratory, the presence of fully mature gE in Pompe cells confirmed that the viral glycoprotein was properly processed in the *trans*-Golgi network, where the final sugar residues are added (43).

## DISCUSSION

The extreme cell-associated nature of VZV infection has been a subject of considerable research since the original discovery in 1953 by the Nobel laureate T. H. Weller (44). We now know the importance of an insightful study of alphaherpesvirus envelopment in 1968, which pointed out the fact that enveloped herpesviral particles cluster in small vacuoles and large vacuoles in the cytoplasm. (45). Based on extensive imaging of VZV infection in Pompe cells, the underlying conclusion of this report is that the small and large vacuoles represent two distinguishable pathways of egress for VZV particles after secondary envelopment. The discovery of one of the two pathways was dependent on our prior VZV studies of autophagy and autophagic flux (17). Autophagy is an ancient cellular process (46). Herpesviruses have coevolved in cells of their hosts since ancient times (47). We have proposed that an intact autophagy pathway leads to greater recovery of infectivity during the VZV infectious cycle (3). The VZV genome is the smallest among the human herpesviruses; it does not code for a homolog of the HSV1 neurovirulence protein ICP34.5, which binds to Beclin1 and thereby inhibits autophagy (48, 49). Based on multiple reports over the past decades, the most common VZV egress pathway in cultured cells involves a vacuole in the autophagic flux pathway, namely, the late endosome. This late endosome pathway includes the M6PR, where most of the M6PRs reside (50, 51). In this report, we provide evidence that the late endosome compartment containing the viral particles also contains the M6PRs. The alternative or second VZV egress pathway does not involve the late endosome or the M6PR. Although both pathways transport infectious VZV particles, based on data in Fig. 1 about viral titers in MRC-5 cells versus Pompe cells, the production of infectious particles in Pompe cells was markedly reduced.

The conclusions within this report were reliant on an informative series of electron micrographs. We have periodically observed VZV-infected cells by electron microscopy for decades in this research laboratory (20, 26, 29, 43, 52). In one set of experiments, we examined VZV-infected cells by electron microscopy at early time points of 15 min, 8 h, 20 h, and 30 h after infection to document the minimal presence of inoculum virus. Therefore, we have an archive of over 1,000 electron micrographs, some on photographic paper and, more recently, in digital format (8). For this new research project, we observed an additional 483 digital electron micrographs of VZV-infected cells. In comparisons with VZV-infected MRC-5 cells or melanoma cells, we quickly noticed differences in the VZV-infected Pompe cells. The most important difference was the virtual absence of the large cytoplasmic vacuoles containing numerous VZV particles, often a mixture of aberrant enveloped capsids, light particles, and a few prototypic virions. The Gershon laboratory has described the large single-walled vacuoles containing VZV particles as late endosomes and lysosomes (39); we have described the large vacuoles as late endosomes or amphisomes (36). Because the M6PR is considered a marker of late endosomes, we have decided to label the pathway as the M6PR pathway rather than choosing a name of a particular organelle. The absence of large vacuoles with viral particles in VZV-infected Pompe cells strongly suggested that trafficking of viral particles to late endosomes was reduced, thus limiting the formation of the vacuoles. We did not find any sorting of viral particles to a lysosome in Pompe cells. Likewise, in MRC-5 cells, we have not found any sorting of viral particles from a late endosome to a lysosome; similarly, the Johnson laboratory never found any sorting of HSV1 particles to a lysosome (38).

A reduction in large vacuoles containing viral particles had previously been observed in human melanoma cells in which the formation of M6PRs was suppressed (39). M6PRs bind to M6P groups on newly formed lysosomal hydrolases in the TGN and facilitate the packaging of the hydrolases into vesicles that are eventually carried to the late endosomes (53). This process is called the M6P-dependent pathway. The enzyme GAA is a glycoprotein with M6P residues that attach to the M6PR. Similarly, some VZV glycoproteins are phosphorylated as they are processed through the Golgi cisternae (54–57). We have confirmed by confocal microscopy a colocalization between a known phosphorylated VZV glycoprotein, gE, and the M6PR in infected MRC-5 cells. Based on the confocal microscopy experiments, we concluded that enveloped VZV particles located within late endosomes remain attached to the M6PRs at the plasma membranes of infected MRC-5 and melanoma cells. This important finding was confirmed through the use of (i) two different anti-M6PR antibodies, produced and characterized independently by two different laboratories, together with (ii) the murine monoclonal antibody to gE, 3B3, which has a relatively high equilibrium dissociation constant (*K_D_*) to its defined linear epitope (*K_D_* = 1.5 × 10^−7^ M) (58). No cross-reactivities of monoclonal antibody (MAb) 3B3 have been defined during its extensive usage since it production in 1983 (59).

Late endosomes comprise large vacuoles (>1,000 nm in diameter) because they can fuse with one another or with other organelles to form hybrid compartments, sometimes called kiss-and-run fusions (60). We had previously called these vacuoles amphisomes (36), but perhaps that designation was overly controversial. We note in our defense that the Seglen laboratory published a specific protocol for purification of amphisomes, and they noted in the characterization of its constituent proteins that amphisomes were enriched in the M6PR (61). They state that “the fact that amphisomes (but not autophagosomes or lysosomes) contain the M6PR, generally regarded as a marker of late endosomes, suggests that amphisomes have undergone fusion with late endosomes.” They also point out that amphisomes frequently contained small fragments of cytoplasm as cargo. We point out the striking similarity between the micrograph of an amphisome shown in Fig. 6F in reference 61 by the Seglen laboratory and the micrograph of a vacuole carrying VZV particles as well as cytoplasmic fragments in Fig. 5A2. We speculate that the short external cytoplasmic tails of the M6PRs housed within the large vacuoles, which are known to contain the signals to recognize kinesin-3 motor proteins, direct the vacuole with its viral cargo to the plasma membrane (62).

VZV exocytosis in the small vacuole pathway in Pompe cells is the alternative pathway that does not involve the M6PR. When we purified viral particles from Pompe cells by density gradient sedimentation, we were able to detect both the VZV gE protein and the Rab6 protein in the virus band. Features of this secretory pathway have been described by the Elliott laboratory and the Enquist laboratory, using HSV1 and PRV, respectively (63). Both laboratories used Rab6 as a marker for the transport vesicle (35, 64). Further, the Enquist laboratory has shown that a kinesin-3 recruitment complex facilitates trafficking of an enveloped PRV or HSV1 particle within an axon in the rat superior cervical ganglion (65).

**FIG 10:**
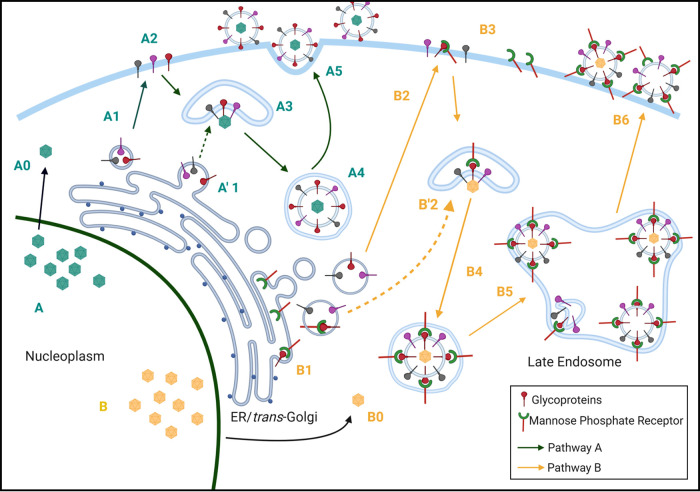
Egress pathways of VZV. We propose two major egress pathways based on data from ourselves and others cited in Results. One pathway has been named the small vacuole pathway or Rab6 pathway, based on data mainly from PRV and HSV1 studies (green arrows). The other pathway has been named the large vacuole pathway or M6PR pathway, based on data mainly from VZV and HSV1 studies (yellow arrows). For each of the two major pathways, there are two entry routes by which VZV glycoproteins can travel from the site of biosynthesis in the Golgi apparatus/TGN to the VAC (wrapping compartment). In this figure, the assumption has been made that the VAC is derived from the TGN. In the first route, viral glycoproteins are transported to the plasma membrane from the TGN and then undergo endocytosis with trafficking to the VAC; in the second route, the VAC is derived directly from viral glycoproteins within an extension from the TGN. (A) In the small vacuole pathway, capsids are green (A0). Capsids exit the nucleus. Either viral glycoproteins are transported to the plasma membrane (A1, A2) and internalized or small glycoprotein-containing vacuoles themselves (A′1) act as a VAC (A3), after which virions with viral envelopes lacking the M6PR are transported in a small vacuole (A4) directly to the plasma membrane (A5). (B) In the large vacuole pathway, capsids are yellow (B0). Capsids exit the nucleus. Either viral glycoproteins are transported to the plasma membrane (B1, B2), where they are recognized by the M6PR and internalized as a glycoprotein/M6PR complex (B3), or viral glycoproteins are attached to the M6PR in the TGN (B1) and the glycoprotein/M6PR complex evolves into the VAC (B′2). After exiting the VAC (B4), virions with M6P-positive glycoproteins still attached to M6PRs are transported to the late endosome (B5). Fusion of these vacuoles to a late endosome leads to a large M6PR-positive vacuole with multiple viral particles. In turn, signals in the cytoplasmic tails of the M6PRs facilitate transport of a late endosome with its viral cargo to the plasma membrane (B6), where M6PR and VZV gE colocalization was detectable by confocal microscopy (Fig. 7 and 8). Note the presence of light particles in pathway B. In contrast, M6PR/gE colocalization was not detectable on the surfaces of VZV-infected Pompe cells (pathway A).

Finally, we present in Fig. 10 an update of our earlier model of virus egress that included two routes of egress from the virus assembly compartment (36). However, the role of the M6PR in one egress pathway did not become apparent until we performed the current experiments in autophagy-deficient Pompe cells, in which the M6PR pathway is essentially blocked (23). Most investigators consider the TGN to be the source of the virus assembly compartment, probably the same structure as the wrapping compartment (5). The viral glycoproteins can travel directly to the VAC, or they can travel to the plasma membrane, where they undergo endocytosis and then travel to the VAC (66). Likewise, the M6PR can attach to viral glycoproteins either in the TGN or on the cell surface (23, 50). Under either scenario, envelopment occurs in the VAC and enveloped virions without the M6PR travel directly to the plasma membrane in small vacuoles (35, 63). As shown in this report, viral particles with M6P residues in their envelope glycoproteins are transported in the M6PR pathway to a late endosome. In turn, the late endosome containing a cargo of several particles, still attached to M6PRs, are transported to the plasma membrane without undergoing xenophagy. Since glycoproteins containing M6P residues appear to be incompletely processed glycoproteins, viral particles containing these glycoproteins in their envelope may be less infectious, an explanation for the invariably low titer of VZV grown in cultured cells (37, 56).

The interaction between the M6PR and gE on the viral particle may have been easier to recognize in our experiments because they were observed within the distinctive viral highways on the cell surface by high-resolution confocal microscopy (29). We have now renamed the pathways; pathway A is the Rab6 or small vacuole pathway, and pathway B is the M6PR or large vacuole pathway (Fig. 10). The Rab6 secretory pathway outlined by the Enquist laboratory for PRV and the Elliott laboratory for HSV1 is probably the M6PR-independent pathway proposed by the Johnson laboratory (38). Based upon the preceding review of the PRV and HSV1 literature, we propose that the M6PR pathway is most utilized in VZV infection and least utilized in HSV1 infection, with PRV’s usage being closer to HSV1’s. The presence of viral highways support that conclusion, since they are seen only after VZV infection of cultured cells and not after HSV1 or PRV infection (26). Although we have not performed any TEM studies on VZV-infected neuronal tissues, the Arvin laboratory has examined human dorsal root ganglia by TEM after VZV infection in their severe combined immunodeficient mouse model. Of great relevance to this report, they observed several large cytoplasmic vacuoles filled with aberrant viral particles in the satellite cells; these vacuoles probably represent the M6PR pathway of virus trafficking shown in Fig. 5A (67).

There may also be an evolutionary link between the M6PR and HSV1 and VZV glycoproteins. Viral genes within the unique short genome (including both gD and gE) are considered to be late additions to each alphaherpesvirus genome that facilitate and fine-tune adaption to its specific host. There is speculation that the predominant VZV gE protein (VZV ORF 68; 623 amino acids; contains M6P residues), which is larger than its HSV1 gE counterpart (HSV1 US8; 552 amino acids; no M6P residues), has subsumed functions of the small HSV1 gD (HSV1 US6; 369 amino acids; contains M6P residues). Even though VZV and PRV are more closely related to each other than to HSV1, VZV has lost gD, while PRV has retained gD (25, 68). Together, these results suggest that the absence of a gD homolog may influence the pathway selected by VZV during egress.

## MATERIALS AND METHODS

### Virus, cells, and conditions of infection.

The VZV strain was VZV-32, a low-passage-number laboratory strain that has been completely sequenced (GenBank accession number DQ479961) (69). Adenovirus serotype 35 and a rabbit antihexon antibody were obtained from M. S. Horwitz (Albert Einstein College of Medicine) (70, 71). Viruses were grown in MRC-5 fibroblast cells (ATCC), human melanoma cells (strain MeWo; Sloan Kettering Cancer Center), or cells obtained from a child with Pompe disease (33). Pompe disease cells were obtained from the Coriell Institute (accession number GM00244). These cells are fibroblasts that were collected from a 5-month-old infant with fatal disease. The organ from which the Pompe fibroblasts were collected is not recorded. Pompe cell monolayers were grown in minimum essential medium (MEM) containing 15% fetal calf serum; they were incubated at 37°C in a humidified incubator. This lineage is not transformed; the cells can be passaged at least 4 to 6 times. The conditions of infection of Pompe cell monolayers were the same as for MRC-5 or melanoma cell monolayers, with selection of either an infected MRC-5 cell inoculum (10^5^ infectious units/ml) or cell-free MRC-5 virus inoculum (10^4^ PFU/ml) (72). The inoculum was layered over an existent Pompe cell monolayer; uninfected Pompe cells were not cocultivated with infected MRC-5 cells. Some cultures were incubated in the presence of the autophagy inhibitor BAF (Sigma; B1793), as described previously (8). Altogether, over 30 experiments have been carried out in Pompe cells. The conditions for VZV infectious center assays and VZV plaque assays have been described (33). To measure the titer of cell-free virus in medium overlying infected Pompe cells, we collected the medium when the cytopathic effect was evident in the monolayer, sedimented the medium to remove cellular debris, and then titrated the medium in 24-well plates. To measure the titer of cell-free virus in monolayers of infected MRC-5 cells or Pompe cells, we dislodged the infected monolayer with a rubber policeman, sedimented the cells into a pellet, and sonicated the pellet. After a low-speed sedimentation to remove debris, the sonicate was titrated in 24-well plates. The viral titers represent means and standard errors of the means from three independent experiments.

### Antibody reagents.

Monoclonal antibodies against VZV proteins were produced and characterized at the University of Iowa, Iowa City, IA, USA (73). Rabbit antibody to LC3 was obtained from Sigma (L7543); antibody to LAMP1 (H4A3) and antibody to LAMP2 (H4B4) were obtained from the Developmental Studies Hybridoma Bank, NIH. Rab6 antibody was obtained from ThermoFisher Scientific (PA5-22127). Two antibodies against the M6PR were produced independently by different research laboratories. Rabbit mono-specific antibody against the human M6PR was produced and characterized by the C. Scott laboratory at the Kolling Institute of Medical Research, St. Leonard’s, Australia (40). This antibody recognized mainly CI-M6PR. A second mono-specific rabbit antibody against the human M6PR was produced and characterized by the D. Johnson laboratory at the Oregon Health Sciences University (41).This antibody recognized both CI-M6PR and CD-M6PR. The first anti-M6PR antibody was produced in order to perform M6PR experiments in cancer biology and therefore is a control for the second anti-M6PR antibody, which was made to investigate the role of M6PR interactions with gD in HSV1 infection (38, 74).

### Imaging by confocal microscopy, transmission electron microscopy, and scanning electron microscopy.

The methods for analysis of VZV-infected monolayers with a Zeiss 710 confocal microscope have been described in detail by this laboratory (75). The following fluoroprobes were obtained from Life Technologies: Alexa Fluor 546 (A11018), Alexa Fluor 488 (A11070), and Alexa Fluor 488 (A11017). The use of Imaris software for converting z-stacks of 2D images into 3D animations has been described by this laboratory, but the methods for this project included newer software (Oxford Instruments). Confocal z-stacks were visualized with Imaris 3/4D Image Visualization and Analysis software after file conversion using the Imaris Converter app. Isosurface renderings of the nuclei were generated for spatial reference. The Imaris Coloc module was used to identify virus and receptor binding. The areas of colocalization were displayed in magenta as both isosurface renderings and maximum-intensity projections, which represents the level of maximum intensity along the *z* axis for each *x*,*y* position. The methods for scanning electron microscopy of VZV-infected monolayers have been described in detail in prior publications (76). The methods for electron microscopy were described also in articles from this laboratory (8, 29). For this study, a total of 483 new electron micrographs were viewed. As noted in an earlier study, we have a total of 1,152 archived electron micrographs (8). In our descriptions of cytoplasmic compartments, we have used the words “small” and “large” vacuoles. Because the diameter of the small vacuoles is usually <250 nm, these could have been called “vesicles” also.

### Purification of VZV by density viscosity gradient sedimentation.

The conditions for purification of VZV light particles and enveloped infectious particles by density viscosity gradient sedimentation have been described in detail in prior publications (72). Adenovirus was purified in the same gradients.

### Immunoblotting.

Techniques for immunoblotting of viral and many cellular proteins have been described by this laboratory (77). In addition, we reviewed techniques for immunoblotting the Rab6 protein by other laboratories (78). Electrophoresis was performed in Bio-Rad mini-protean 4 to 20% precast polyacrylamide gels.

### RNA studies.

Total RNA was extracted from uninfected and VZV-infected fibroblasts or Pompe cells in six-well plates at the given time points using the RNeasy minikit (Qiagen). RNA quality and quantity were assayed by UV spectroscopy using a NanoDrop spectrometer (ThermoFisher Scientific). *A*_260_/*A*_280_ measured ratios were within 20% of 2.0, and infected cells from a six-well plate well (6.5 cm^2^) yielded approximately 3 μg of RNA in 80 μl. Polyadenylated RNA was converted to cDNA using anchored oligo(dT) primers and the SuperScript III first-strand synthesis system for RT-PCR (Invitrogen), to yield approximately 20 ng of cDNA. The samples were subsequently digested with ribonuclease H to remove RNA bound to the cDNA. One microliter of cDNA was added to 25 μl of Applied Biosystems master mix-water, together with approximately 200 nM specific primers (see the primer table in Fig. 9). RT-PCR measurements were carried out using the QuantStudio Flex real-time PCR machine (Applied Biosystems). The resulting PCR results were processed using the QuantStudio real-time PCR software (Applied Biosystems). Threshold cycle (*C_T_*) values of duplicate samples were averaged and normalized to an average of 16.0 for GAPDH (glyceraldehyde-3-phosphate dehydrogenase) to form Δ*C_T_* values. Subsequent ΔΔ*C_T_* values were calculated by differences between averages of VZV-infected Δ*C_T_* values with the average uninfected Δ*C_T_* values. Data points represent means and standard errors of the means from three independent experiments.

## References

[1] Dong X, Levine B 2013 Autophagy and viruses: adversaries or allies? J Innate Immun 5:480–493. doi:10.1159/000346388. 23391695 PMC3790331

[2] Jackson WT 2015 Viruses and the autophagy pathway. Virology 479-480:450–456. doi:10.1016/j.virol.2015.03.042. 25858140 PMC5917100

[3] Grose C, Buckingham EM, Jackson W, Carpenter JE 2015 The pros and cons of autophagic flux among herpesviruses. Autophagy 11:716–717. doi:10.1080/15548627.2015.1017223. 25905782 PMC4502768

[4] Choi Y, Bowman JW, Jung JU 2018 Autophagy during viral infection—a double-edged sword. Nat Rev Microbiol 16:341–354. doi:10.1038/s41579-018-0003-6. 29556036 PMC6907743

[5] Crump C 2018 Virus assembly and egress of HSV. Adv Exp Med Biol 1045:23–44. doi:10.1007/978-981-10-7230-7_2. 29896661

[6] Vinod V, Padmakrishnan CJ, Vijayan B, Gopala S 2014 ‘How can I halt thee?’ The puzzles involved in autophagic inhibition. Pharmacol Res 82:1–8. doi:10.1016/j.phrs.2014.03.005. 24657238

[7] Harley CA, Dasgupta A, Wilson DW 2001 Characterization of herpes simplex virus-containing organelles by subcellular fractionation: role for organelle acidification in assembly of infectious particles. J Virol 75:1236–1251. doi:10.1128/JVI.75.3.1236-1251.2001. 11152497 PMC114030

[8] Girsch JH, Walters K, Jackson W, Grose C 2019 Progeny Varicella-Zoster virus capsids exit the nucleus but never undergo secondary envelopment during autophagic flux inhibition by bafilomycin A1. J Virol 93:e00505-19. doi:10.1128/JVI.00505-19. 31217243 PMC6694825

[9] Prentice E, Jerome WG, Yoshimori T, Mizushima N, Denison MR 2004 Coronavirus replication complex formation utilizes components of cellular autophagy. J Biol Chem 279:10136–10141. doi:10.1074/jbc.M306124200. 14699140 PMC7957857

[10] Wong J, Zhang J, Si X, Gao G, Mao I, McManus BM, Luo H 2008 Autophagosome supports coxsackievirus B3 replication in host cells. J Virol 82:9143–9153. doi:10.1128/JVI.00641-08. 18596087 PMC2546883

[11] Buckingham EM, Carpenter JE, Jackson W, Grose C 2014 Autophagy and the effects of its inhibition on varicella-zoster virus glycoprotein biosynthesis and infectivity. J Virol 88:890–902. doi:10.1128/JVI.02646-13. 24198400 PMC3911683

[12] Xu C, Wang M, Song Z, Wang Z, Liu Q, Jiang P, Bai J, Li Y, Wang X 2018 Pseudorabies virus induces autophagy to enhance viral replication in mouse neuro-2a cells in vitro. Virus Res 248:44–52. doi:10.1016/j.virusres.2018.02.004. 29452162

[13] Kohler L, Puertollano R, Raben N 2018 Pompe disease: from basic science to therapy. Neurotherapeutics 15:928–942. doi:10.1007/s13311-018-0655-y. 30117059 PMC6277280

[14] Hers HG 1963 α-Glucosidase deficiency in generalized glycogen storage disease (Pompe’s disease). Biochem J 86:11–16. doi:10.1042/bj0860011. 13954110 PMC1201703

[15] Raben N, Baum R, Schreiner C, Takikita S, Mizushima N, Ralston E, Plotz P 2009 When more is less: excess and deficiency of autophagy coexist in skeletal muscle in Pompe disease. Autophagy 5:111–113. doi:10.4161/auto.5.1.7293. 19001870 PMC3257549

[16] Nascimbeni AC, Fanin M, Angelini C, Sandri M 2017 Autophagy dysregulation in Danon disease. Cell Death Dis 8:e2565. doi:10.1038/cddis.2016.475. PMC5386379 28102838

[17] Buckingham EM, Carpenter JE, Jackson W, Zerboni L, Arvin AM, Grose C 2015 Autophagic flux without a block differentiates varicella-zoster virus infection from herpes simplex virus infection. Proc Natl Acad Sci U S A 112:256–261. doi:10.1073/pnas.1417878112. 25535384 PMC4291665

[18] Gershon A, Cosio L, Brunell PA 1973 Observations on the growth of varicella-zoster virus in human diploid cells. J Gen Virol 18:21–31. doi:10.1099/0022-1317-18-1-21. 4354535

[19] Almeida JD, Howatson AF, Williams MG 1962 Morphology of varicella (chicken pox) virus. Virology 16:353–355. doi:10.1016/0042-6822(62)90261-1. 13860662

[20] Jones F, Grose C 1988 Role of cytoplasmic vacuoles in varicella-zoster virus glycoprotein trafficking and virion envelopment. J Virol 62:2701–2711. doi:10.1128/JVI.62.8.2701-2711.1988. 2839696 PMC253703

[21] Graybill C, Morgan MJ, Levin MJ, Lee KS 2018 Varicella-zoster virus inhibits autophagosome-lysosome fusion and the degradation stage of mTOR-mediated autophagic flux. Virology 522:220–227. doi:10.1016/j.virol.2018.07.018. 30053655

[22] Takahashi MN, Jackson W, Laird DT, Culp TD, Grose C, Haynes JI II, Benetti L 2009 Varicella-zoster virus infection induces autophagy in both cultured cells and human skin vesicles. J Virol 83:5466–5476. doi:10.1128/JVI.02670-08. 19297471 PMC2681990

[23] Cardone M, Porto C, Tarallo A, Vicinanza M, Rossi B, Polishchuk E, Donaudy F, Andria G, De Matteis MA, Parenti G 2008 Abnormal mannose-6-phosphate receptor trafficking impairs recombinant alpha-glucosidase uptake in Pompe disease fibroblasts. Pathogenetics 1:6. doi:10.1186/1755-8417-1-6. 19046416 PMC2635360

[24] Fukuda T, Ewan L, Bauer M, Mattaliano RJ, Zaal K, Ralston E, Plotz PH, Raben N 2006 Dysfunction of endocytic and autophagic pathways in a lysosomal storage disease. Ann Neurol 59:700–708. doi:10.1002/ana.20807. 16532490

[25] Grose C 2002 The predominant varicella-zoster virus gE and gI glycoprotein complex, p 195–223. *In* Holzenburg A, Bogner E (ed), Structure-function relationships of human pathogenic viruses. Kluwer Academic Press, New York, NY.

[26] Padilla JA, Nii S, Grose C 2003 Imaging of the varicella zoster virion in the viral highways: comparison with herpes simplex viruses 1 and 2, cytomegalovirus, pseudorabies virus, and human herpes viruses 6 and 7. J Med Virol 70:S103–S110. doi:10.1002/jmv.10330. 12627497

[27] Storlie J, Carpenter JE, Jackson W, Grose C 2008 Discordant varicella-zoster virus glycoprotein C expression and localization between cultured cells and human skin vesicles. Virology 382:171–181. doi:10.1016/j.virol.2008.09.031. 18954885 PMC2754791

[28] Jahreiss L, Menzies FM, Rubinsztein DC 2008 The itinerary of autophagosomes: from peripheral formation to kiss-and-run fusion with lysosomes. Traffic 9:574–587. doi:10.1111/j.1600-0854.2008.00701.x. 18182013 PMC2329914

[29] Harson R, Grose C 1995 Egress of varicella-zoster virus from the melanoma cell: a tropism for the melanocyte. J Virol 69:4994–5010. doi:10.1128/JVI.69.8.4994-5010.1995. 7609070 PMC189316

[30] Grose C, Harson R, Beck S 1995 Computer modeling of prototypic and aberrant nucleocapsids of varicella-zoster virus. Virology 214:321–329. doi:10.1006/viro.1995.0041. 8553532

[31] Nii S, Morgan C, Rose HM 1968 Electron microscopy of herpes simplex virus. II. Sequence of development. J Virol 2:517–536. doi:10.1128/JVI.2.5.517-536.1968. 4301317 PMC375641

[32] Gershon AA, Sherman DL, Zhu Z, Gabel CA, Ambron RT, Gershon MD 1994 Intracellular transport of newly synthesized varicella-zoster virus: final envelopment in the trans-Golgi network. J Virol 68:6372–6390. doi:10.1128/JVI.68.10.6372-6390.1994. 8083976 PMC237058

[33] Grose C, Brunel PA 1978 Varicella-zoster virus: isolation and propagation in human melanoma cells at 36 and 32 degrees C. Infect Immun 19:199–203. doi:10.1128/IAI.19.1.199-203.1978. 203532 PMC414067

[34] Hogue IB, Bosse JB, Hu JR, Thiberge SY, Enquist LW 2014 Cellular mechanisms of alpha herpesvirus egress: live cell fluorescence microscopy of pseudorabies virus exocytosis. PLoS Pathog 10:e1004535. doi:10.1371/journal.ppat.1004535. 25474634 PMC4256261

[35] Hogue IB, Scherer J, Enquist LW 2016 Exocytosis of alphaherpesvirus virions, light particles, and glycoproteins uses constitutive secretory mechanisms. mBio 7:e00820-16. doi:10.1128/mBio.00820-16. 27273828 PMC4959669

[36] Buckingham EM, Jarosinski KW, Jackson W, Carpenter JE, Grose C 2016 Exocytosis of varicella-zoster virus virions involves a convergence of endosomal and autophagy pathways. J Virol 90:8673–8685. doi:10.1128/JVI.00915-16. 27440906 PMC5021422

[37] Brunetti CR, Burke RL, Kornfeld S, Gregory W, Masiarz FR, Dingwell KS, Johnson DC 1994 Herpes simplex virus glycoprotein D acquires mannose 6-phosphate residues and binds to mannose 6-phosphate receptors. J Biol Chem 269:17067–17074. 8006011

[38] Brunetti CR, Dingwell KS, Wale C, Graham FL, Johnson DC 1998 Herpes simplex virus gD and virions accumulate in endosomes by mannose 6-phosphate-dependent and -independent mechanisms. J Virol 72:3330–3339. doi:10.1128/JVI.72.4.3330-3339.1998. 9525660 PMC109812

[39] Chen JJ, Zhu Z, Gershon AA, Gershon MD 2004 Mannose 6-phosphate receptor dependence of varicella zoster virus infection in vitro and in the epidermis during varicella and zoster. Cell 119:915–926. doi:10.1016/j.cell.2004.11.007. 15620351

[40] Costello M, Baxter RC, Scott CD 1999 Regulation of soluble insulin-like growth factor II/mannose 6-phosphate receptor in human serum: measurement by enzyme-linked immunosorbent assay. J Clin Endocrinol Metab 84:611–617. doi:10.1210/jcem.84.2.5488. 10022425

[41] Brunetti CR, Burke RL, Hoflack B, Ludwig T, Dingwell KS, Johnson DC 1995 Role of mannose-6-phosphate receptors in herpes simplex virus entry into cells and cell-to-cell transmission. J Virol 69:3517–3528. doi:10.1128/JVI.69.6.3517-3528.1995. 7745699 PMC189065

[42] Grose C 1990 Glycoproteins encoded by varicella-zoster virus: biosynthesis, phosphorylation, and intracellular trafficking. Annu Rev Microbiol 44:59–80. doi:10.1146/annurev.mi.44.100190.000423. 2174668

[43] Montalvo EA, Parmley RT, Grose C 1985 Structural analysis of the varicella-zoster virus gp98-gp62 complex: posttranslational addition of N-linked and O-linked oligosaccharide moieties. J Virol 53:761–770. doi:10.1128/JVI.53.3.761-770.1985. 2983087 PMC254704

[44] Weller TH 1953 Serial propagation in vitro of agents producing inclusion bodies derived from varicella and herpes zoster. Proc Soc Exp Biol Med 83:340–346. doi:10.3181/00379727-83-20354. 13064265

[45] Darlington RW, Moss LH III 1968 Herpesvirus envelopment. J Virol 2:48–55. doi:10.1128/JVI.2.1.48-55.1968. 4316013 PMC375577

[46] Klionsky DJ 2013 Ancient autophagy. Autophagy 9:445–446. doi:10.4161/auto.23907. 23388466 PMC3627661

[47] McGeoch DJ, Davison AJ 1999 The molecular evolutionary history of the herpesviruses, p 441–465. *In* Domingo E, Webster R, Holland J (ed), Origin and evolution of viruses. Academic Press, New York, NY.

[48] Davison AJ, Scott JE 1986 The complete DNA sequence of varicella-zoster virus. J Gen Virol 67:1759–1816. doi:10.1099/0022-1317-67-9-1759. 3018124

[49] Orvedahl A, Alexander D, Talloczy Z, Sun Q, Wei Y, Zhang W, Burns D, Leib DA, Levine B 2007 HSV-1 ICP34.5 confers neurovirulence by targeting the Beclin 1 autophagy protein. Cell Host Microbe 1:23–35. doi:10.1016/j.chom.2006.12.001. 18005679

[50] Kornfeld S 1992 Structure and function of the mannose 6-phosphate/insulinlike growth factor II receptors. Annu Rev Biochem 61:307–330. doi:10.1146/annurev.bi.61.070192.001515. 1323236

[51] Stoorvogel W, Strous GJ, Geuze HJ, Oorschot V, Schwartz AL 1991 Late endosomes derive from early endosomes by maturation. Cell 65:417–427. doi:10.1016/0092-8674(91)90459-C. 1850321

[52] Grose C 1996 Pathogenesis of infection with varicella vaccine. Infect Dis Clin North Am 10:489–505. doi:10.1016/S0891-5520(05)70310-X. 8856349

[53] Coutinho MF, Prata MJ, Alves S 2012 Mannose-6-phosphate pathway: a review on its role in lysosomal function and dysfunction. Mol Genet Metab 105:542–550. doi:10.1016/j.ymgme.2011.12.012. 22266136

[54] Montalvo EA, Grose C 1986 Varicella zoster virus glycoprotein gpI is selectively phosphorylated by a virus-induced protein kinase. Proc Natl Acad Sci U S A 83:8967–8971. doi:10.1073/pnas.83.23.8967. 3024158 PMC387055

[55] Edson CM, Hosler BA, Waters DJ 1987 Varicella-zoster virus gpI and herpes simplex virus gE: phosphorylation and Fc binding. Virology 161:599–602. doi:10.1016/0042-6822(87)90157-7. 2825425

[56] Gabel CA, Dubey L, Steinberg SP, Sherman D, Gershon MD, Gershon AA 1989 Varicella-zoster virus glycoprotein oligosaccharides are phosphorylated during posttranslational maturation. J Virol 63:4264–4276. doi:10.1128/JVI.63.10.4264-4276.1989. 2550667 PMC251041

[57] Grose C 1991 Glycoproteins of varicella-zoster virus and their herpes simplex homologs. Rev Infect Dis 13:S960–S963. doi:10.1093/clind/13.Supplement_11.S960. 1664135

[58] Grose C, Tyler S, Peters G, Hiebert J, Stephens GM, Ruyechan WT, Jackson W, Storlie J, Tipples GA 2004 Complete DNA sequence analyses of the first two varicella-zoster virus glycoprotein E (D150N) mutant viruses found in North America: evolution of genotypes with an accelerated cell spread phenotype. J Virol 78:6799–6807. doi:10.1128/JVI.78.13.6799-6807.2004. 15194755 PMC421634

[59] Halling G, Giannini C, Britton JW, Lee RW, Watson RE Jr, Terrell CL, Parney IF, Buckingham EM, Carpenter JE, Grose C 2014 Focal encephalitis following varicella-zoster virus reactivation without rash in a healthy immunized young adult. J Infect Dis 210:713–716. doi:10.1093/infdis/jiu137. 24604820 PMC4202304

[60] Huotari J, Helenius A 2011 Endosome maturation. EMBO J 30:3481–3500. doi:10.1038/emboj.2011.286. 21878991 PMC3181477

[61] Berg TO, Fengsrud M, Stromhaug PE, Berg T, Seglen PO 1998 Isolation and characterization of rat liver amphisomes. Evidence for fusion of autophagosomes with both early and late endosomes. J Biol Chem 273:21883–21892. doi:10.1074/jbc.273.34.21883. 9705327

[62] Ara A, Ahmed KA, Xiang J 2018 Mannose-6-phosphate receptor: a novel regulator of T cell immunity. Cell Mol Immunol 15:986–988. doi:10.1038/s41423-018-0031-1. 29769659 PMC6207697

[63] Hollinshead M, Johns HL, Sayers CL, Gonzalez-Lopez C, Smith GL, Elliott G 2012 Endocytic tubules regulated by Rab GTPases 5 and 11 are used for envelopment of herpes simplex virus. EMBO J 31:4204–4220. doi:10.1038/emboj.2012.262. 22990238 PMC3492727

[64] Johns HL, Gonzalez-Lopez C, Sayers CL, Hollinshead M, Elliott G 2014 Rab6 dependent post-Golgi trafficking of HSV1 envelope proteins to sites of virus envelopment. Traffic 15:157–178. doi:10.1111/tra.12134. 24152084 PMC4345966

[65] Scherer J, Hogue IB, Yaffe ZA, Tanneti NS, Winer BY, Vershinin M, Enquist LW 2020 A kinesin-3 recruitment complex facilitates axonal sorting of enveloped alpha herpesvirus capsids. PLoS Pathog 16:e1007985. doi:10.1371/journal.ppat.1007985. 31995633 PMC7010296

[66] Maresova L, Pasieka TJ, Homan E, Gerday E, Grose C 2005 Incorporation of three endocytosed varicella-zoster virus glycoproteins, gE, gH, and gB, into the virion envelope. J Virol 79:997–1007. doi:10.1128/JVI.79.2.997-1007.2005. 15613328 PMC538533

[67] Reichelt M, Zerboni L, Arvin AM 2008 Mechanisms of varicella-zoster virus neuropathogenesis in human dorsal root ganglia. J Virol 82:3971–3983. doi:10.1128/JVI.02592-07. 18256143 PMC2292995

[68] Carfi A, Gong H, Lou H, Willis SH, Cohen GH, Eisenberg RJ, Wiley DC 2002 Crystallization and preliminary diffraction studies of the ectodomain of the envelope glycoprotein D from herpes simplex virus 1 alone and in complex with the ectodomain of the human receptor HveA. Acta Crystallogr D Biol Crystallogr 58:836–838. doi:10.1107/s0907444902001270. 11976496

[69] Peters GA, Tyler SD, Grose C, Severini A, Gray MJ, Upton C, Tipples GA 2006 A full-genome phylogenetic analysis of varicella-zoster virus reveals a novel origin of replication-based genotyping scheme and evidence of recombination between major circulating clades. J Virol 80:9850–9860. doi:10.1128/JVI.00715-06. 16973589 PMC1617253

[70] Horwitz MS, Valderrama G, Hatcher V, Korn R, deJong P, Spigland I 1984 Characterization of adenovirus isolates from AIDS patients. Ann N Y Acad Sci 437:161–174. doi:10.1111/j.1749-6632.1984.tb37132.x. 6099998

[71] Horwitz MS, Maizel JV Jr, Scharff MD 1970 Molecular weight of adenovirus type 2 hexon polypeptide. J Virol 6:569–571. doi:10.1128/JVI.6.4.569-571.1970. 5497905 PMC376158

[72] Grose C, Perrotta DM, Brunell PA, Smith GC 1979 Cell-free varicella-zoster virus in cultured human melanoma cells. J Gen Virol 43:15–27. doi:10.1099/0022-1317-43-1-15. 225414

[73] Grose C, Edwards DP, Friedrichs WE, Weigle KA, McGuire WL 1983 Monoclonal antibodies against three major glycoproteins of varicella-zoster virus. Infect Immun 40:381–388. doi:10.1128/IAI.40.1.381-388.1983. 6299963 PMC264858

[74] Caixeiro NJ, Martin JL, Scott CD 2013 Silencing the mannose 6-phosphate/IGF-II receptor differentially affects tumorigenic properties of normal breast epithelial cells. Int J Cancer 133:2542–2550. doi:10.1002/ijc.28276. 23686499

[75] Jackson W, Yamada M, Moninger T, Grose C 2013 Visualization and quantitation of abundant macroautophagy in virus-infected cells by confocal three-dimensional fluorescence imaging. J Virol Methods 193:244–250. doi:10.1016/j.jviromet.2013.06.018. 23792686 PMC3735810

[76] Carpenter JE, Hutchinson JA, Jackson W, Grose C 2008 Egress of light particles among filopodia on the surface of varicella-zoster virus-infected cells. J Virol 82:2821–2835. doi:10.1128/JVI.01821-07. 18184710 PMC2258984

[77] Carpenter JE, Jackson W, de Souza GA, Haarr L, Grose C 2010 Insulin-degrading enzyme binds to the nonglycosylated precursor of varicella-zoster virus gE protein found in the endoplasmic reticulum. J Virol 84:847–855. doi:10.1128/JVI.01801-09. 19864391 PMC2798375

[78] Monier S, Goud B 2005 Purification and properties of rab6 interacting proteins. Methods Enzymol 403:593–599. doi:10.1016/S0076-6879(05)03051-X. 16473622

